# Risk reduction and precision prevention across the Alzheimer’s disease continuum: a systematic review of clinical trials combining multidomain lifestyle interventions and pharmacological or nutraceutical approaches

**DOI:** 10.1016/j.tjpad.2025.100367

**Published:** 2025-10-27

**Authors:** Erika Bereczki, Francesca Mangialasche, Mariagnese Barbera, Paola Padilla, Yuko Hara, Howard Fillit, Alina Solomon, Miia Kivipelto

**Affiliations:** a FINGERS Brain Health Institute, Karolinska Vägen 37A, QA32 171 64, Solna, Sweden; b Division of Neurogeriatrics, Center for Alzheimer Research, Department of Neurobiology, Care Sciences and Society, Karolinska Institutet, Visionsgatan 4 171 64, Solna, Sweden; c Division of Clinical Geriatrics, Center for Alzheimer Research, Department of Neurobiology, Care Sciences and Society, Karolinska Institutet, Karolinska Vägen 37A 171 64, Solna, Sweden; d Theme Inflammation and Aging, Medical Unit Aging, Karolinska University Hospital, Karolinska Vägen 37A 171 76, Solna, Sweden; e Department of Neurology, Institute of Clinical Medicine, University of Eastern Finland, Yliopistonranta 1C 70211, Kuopio, Finland; f The Ageing Epidemiology Research Unit, School of Public Health, Imperial College London, London, UK; g Alzheimer’s Drug Discovery Foundation, 57 West 57th St. Suite 904, New York, NY 10019, USA; h Research and Development Unit, Stockholms Sjukhem, Stockholm, Sweden; i Institute of Public Health and Clinical Nutrition, University of Eastern Finland, Kuopio, Finland

**Keywords:** Multidomain lifestyle intervention, Combination therapy, Dementia prevention, Pharmacological approach, Randomized controlled trial

## Abstract

To effectively combat dementia onset and progression, lifestyle-based interventions targeting multiple risk factors and disease mechanisms through a multidomain approach - tailored and implemented early in the disease process - have emerged as promising. Electronic databases and relevant websites (clinicaltrials.gov, euclinicaltrials.eu, PubMed and EMBASE) were systematically searched for randomized controlled trials (RCTs) testing the combination of multidomain lifestyle and pharmacological interventions. Studies were included if 1) lifestyle intervention was multimodal (≥2 domains); 2) it was combined with drugs, supplements, or medical food; 3) the study population was within the Alzheimer’s disease (AD) and related dementias continuum, including cognitively normal individuals at-risk for dementia, people with subjective cognitive decline (SCD), mild cognitive impairment (MCI), or prodromal AD; 4) outcomes included cognitive or dementia-related measure(s), and 5) intervention lasted at least 6 months. Twelve combination RCTs were identified, incorporating 2 to 7 lifestyle domains (physical exercise, cognitive training, dietary guidance, social activities, sleep hygiene, cardiovascular/metabolic risk management, psychoeducation or stress management), combined with pharmacological components (e.g., Omega-3, Tramiprosate, vitamin D, BBH-1001, epigallocatechin gallate, Souvenaid, and metformin). Seven RCTs targeted participants with prodromal AD, MCI or early dementia, five focused on at risk individuals or SCD. Additionally, 2 studies adopted a precision medicine approach by enriching populations with *APOE-ε4* carriers. Findings suggest that well-designed interventions - tailored to the right individuals, implemented at the optimal time - may effectively improve cognition. However, further refinement of the RCT methodology is warranted, for better alignment with the multifaceted nature of dementia prevention and management.

## 1 Introduction

Healthy ageing, preservation of cognitive functioning, and preventing Alzheimer’s disease (AD) and related dementias (ADRD) is a global priority, as effective ADRD therapeutics are still not widely available. Efforts to find effective treatments are focused on identifying new compounds as well as drug repositioning and repurposing that may potentially delay the onset, slow disease progression, or, ultimately, prevent dementia [1]. Despite recent promising results from anti-amyloid β monoclonal antibody[2,3] therapies with Lecanemab and Donanemab showing potential in slowing cognitive decline, current estimates indicate that <30 % of AD patients would be eligible for these therapies [4,5]. Additionally, the failure of numerous pharmacological and non-pharmacological single-domain interventions in AD underscores the limitations of targeting single-disease mechanisms or single risk factors. There is a clear need for strategies addressing multiple risk factors and biological pathways simultaneously, with early intervention in the ADRD continuum being crucial for an optimal preventive effect [6,7]. These new multidomain therapeutic approaches could enhance treatment efficacy and personalize prevention efforts.

Recently, the update of the Lancet Commission on Dementia Prevention, Intervention and Care included 14 modifiable risk factors for dementia (mostly based on observational studies): low education, hearing and vision loss, traumatic brain injury, hypertension, excessive alcohol use, obesity, smoking, depression, social isolation, physical inactivity, air pollution, diabetes, and high level of low-density lipoprotein (LDL) cholesterol levels [8]. Modifying these factors could prevent or delay up to 45 % of dementia cases. The World Health Organization (WHO) guidelines for risk reduction of cognitive decline and dementia, which are based on the synthesis of findings from intervention trials, recommend, among others, physical activity, a healthy balanced diet, cognitive training, as well as management of hypertension, diabetes, and dyslipidemia, for reducing the risk of cognitive decline [9]. The WHO guidelines are currently being updated, and the new edition will include the addition of new modifiable lifestyle factors and multidomain interventions.

Multidomain lifestyle interventions, e.g., based on the Finnish Geriatric Intervention Study to Prevent Cognitive Impairment and Disability (FINGER), reported promising cognitive benefits in older individuals at risk of dementia including[10] in those with genetic susceptibility (*APOE-ε4* carriers) [11,12]. The FINGER randomized controlled trial (RCT) combined 5 components - exercise, cognitive training, social engagement, dietary recommendations and vascular/metabolic risk factor management - in a 2-year intervention [10]. The FINGER study is now a model for similar trials around the globe, with the World-Wide FINGERS (WW-FINGERS) network including 70 member countries and offering a new paradigm to prevent cognitive decline [13].

Given the growing number of modifiable risk factors being identified for late-life dementia, there is a growing need for novel multidomain combination trials. Integrating multidomain lifestyle interventions with pharmacological treatments - whether novel or repurposed - holds promise for advancing precision medicine approaches aimed at preventing cognitive decline and dementia. Existing strategies for cardiovascular and cancer treatment offer useful models for testing combination therapies in ADRD RCTs [7,14].

Over the past decade, combination trials in the ADRD field have been initiated, focusing on combinations of multidomain non-pharmacological lifestyle interventions and pharmacological compounds [[15], [16], [17]]. In this context, we conducted a systematic review to provide a comprehensive overview of such combination RCTs. The review focused on key methodological aspects, such as intervention and trial design, target populations, intervention duration, and adherence. Our aim is to inform the design of future combination RCTs, by identifying common pitfalls and exploring how current and emerging evidence can be leveraged to optimize trial design and guide the development of more effective intervention strategies.

## 2 Methods

### 2.1 Search strategy

We followed the Preferred Reporting Items for Systematic reviews and Meta Analyses (PRISMA) 2020 guidelines [18]. To identify combination therapy RCTs, records in Clinicaltrials.gov and euclinicaltrials.eu databases were searched with the following terms: “Alzheimer`s disease,” “mild cognitive impairment,” “dementia,” “cognitive decline,” combined with either “lifestyle intervention,” “multidomain,” or “multimodal” from inception to May 30, 2025. PubMed and EMBASE were also searched with the same combination of terms in case of omission. All searches were performed independently by E.B. and P.P.

### 2.2 PICO components and inclusion and exclusion criteria

Based on the Population/Intervention/Comparator/Outcomes (PICO) components, the review aimed to identify RCTs targeting adults with normal cognition, or subjective cognitive decline (SCD) or mild cognitive impairment (MCI) or prodromal AD (P); testing lifestyle-based interventions that addressed at least 2 modifiable risk factors for dementia, or included at least 2 intervention components, in combination with additional treatments, such as pharmacological (i.e., drugs), nutraceutical (e.g., medical food, dietary supplements) or the use of non-invasive devices (e.g., transcranial brain stimulation) (I); included appropriate comparators such as placebo, standard of care, lower intensity intervention (C); and assessed the intervention effect on outcomes related to dementia and cognitive impairment (O). RCTs were included regardless of their current status (ongoing, completed, terminated, unknown), or whether published results were available. We excluded RCTs with interventions/treatments that lasted less than 6 months. Studies were also excluded if they included participants with a suspected or known dementia diagnosis, other major neurological or psychiatric disorders (e.g., Parkinson’s disease, stroke; major depression, multiple sclerosis, schizophrenia, bipolar disorder), history of substance abuse, as well as studies focusing on rehabilitation programs for recent cardiovascular, cerebrovascular, respiratory or other medical events, e.g., post-surgery. The search was not limited by date of publication nor geographical location; however, only records or publications in English were included.

### 2.3 Study selection

The Covidence systematic review software was used to manage search results from clinicaltrials.gov, PubMed, and EMBASE. Those derived from euclinicaltrials.eu were manually checked. After duplicates were removed, Title and Abstracts were screened independently by 2 researchers (E.B. and P.P.). The full texts of potentially eligible studies were then obtained and further assessed. Any disagreement on inclusion was resolved by consensus within the research team.

### 2.4 Data extraction

Eligible RCTs had the following data extracted: National Clinical Trial (NCT) number, study title, acronym of the study, current status, number of trial arms, drug component, dose, lifestyle intervention domains, the intervention intensity or modality, primary outcomes, secondary outcomes, trial duration, age for study inclusion, study population (AD continuum), trial sample size, trial timeframe, masking, trial phase, sponsor, and number of sites and countries.

## 3 Results

### 3.1 Characteristics of the multidomain combination RCTs

**Fig. 1:**
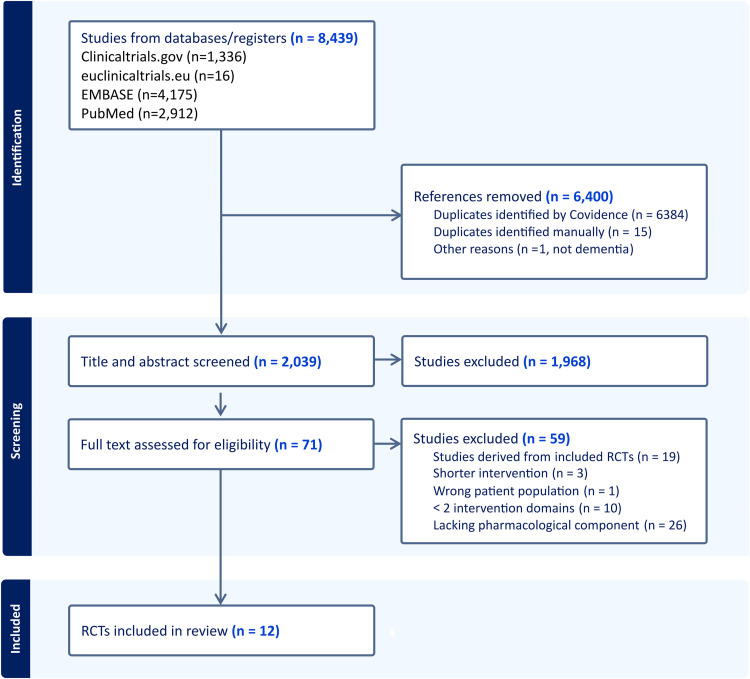
Flow diagram of RCT screening and selection process.

Following our search strategy, 1352 RCTs and 7087 publications were initially identified. After removing duplicates, 2039 publications were screened to assess combinations of lifestyle interventions with pharmacological, nutraceutical or other treatments. This screening process resulted in the identification of 12 combination RCTs (as of 2025.05.30, Fig. 1, Table 1), using several classes of compounds combined with multidomain lifestyle interventions, targeting individuals within the continuum of AD and dementia risk. This included people with normal cognition but increased dementia risk due to risk factors that were not well controlled, identified via validated scores, and people with a diagnosis of SCD, or MCI (defined via different set of diagnostic criteria)[[19], [20], [21]] or prodromal AD. Two RCTs included both MCI and early stage dementia. For some RCTs, participants’ inclusion relied on cognitive screening and assessment of modifiable risk factors (e.g., MET-FINGER, Multidomain Alzheimer Preventive Trial, or MAPT) [15,22]. One trial specifically included participants with MCI who also had concomitant type 2 diabetes (T2D) or insulin resistance, while 2 studies adopted a precision medicine approach by enriching their study populations with individuals carrying the *APOE-ε4* allele (Table 1).

**Table 1:** Clinical trials of multidomain lifestyle interventions combined with pharmacological compounds. RCTs are organized in chronological order. Abbreviations: AD, Alzheimer`s disease; ADAS-Cog, Alzheimer’s Disease Assessment Scale Cognitive; ADCS-PACC, Alzheimer’s Disease Cooperative Study—Preclinical Alzheimer Cognitive Composite; A-IADLQ, Amsterdam Instrumental Activity of Daily Living Questionnaire; AQ, Alzheimer’s Questionnaire; BB, Blood based; CDR, Clinical Dementia Rating; CG, Caregiver; CT, cognitive training; Cv, cardiovascular; D, diet; EGCG, Epigallocatechin gallate, EQ-5D-5L, Euro Quality of Lif—5 dimensions- 5 levels; I, Investigator; LIBRA, Lifestyle for Brain health; M, Meditation; MCI, Mild cognitive impairment; MMSE, Mini mental State Examination; MoCA, Montreal Cognitive Assessment; MRI, Magnetic Resonance Imaging; NA, Not applicable; NTB, Neuropsychological Test Battery; OA, Outcomes assessor; P, Participant; PE, Physical exercise; PsE, Psychoeducation; PROMIS, Patient-reported Outcome Measurement Information System; RAVLT, Rey Auditory Verbal Learning Test; S, Stress management; SA, Social activities; SCWT, Stroop color and word test; SH, Sleep hygiene; SCD, Subjective cognitive decline; T2D, type 2 diabetes; TMT, trail making test. Unless specified, the intervention is delivered by in-person activities, with some RCTs doing intermediate follow-ups also via phone. Trial timeframe covers the entire period from study initiation to completion including recruitment and intervention phases.





* NCT01219244: this study included different dietary interventions (caloric restriction, omega-3 supplementation, resveratrol supplementation) initially tested in single-domain studies, followed by a second step with a combination study including the dietary intervention judged as the most effective, plus physical activity and cognitive training.

** NCT05109169: in this study participants are randomized 1:1 into self-guided (control) vs. structured multimodal lifestyle intervention groups (SMLI). Within the SMLI group, participants who qualify for metformin treatment (elevated adiposity or impaired fasting glucose, but no diabetes) are further randomized 1:1:1 into metformin (2000 mg/day or 1000 mg/day) vs placebo (trial-within-trial).

### 3.2 Duration, frequency and types of intervention domains

A total of 4434 participants were included in the eligible RCTs, with the number of participants ranging from 35 to 1680 (Table 1), and intervention length ranging from 6 to 36 months. Two RCTs had been terminated early as a consequence of the SARS-CoV-2 pandemic (IRMCI and SYNERGIC). Seven RCTs had 2 arms, two RCTs had either 3 or 4 arms, while 1 RCT featured 5 study arms (Table 1). Two studies opted for quadruple masking (P—participants, CG—caregiver, I—investigator, OA—outcome assessor), 3 for triple masking (P, OA, and either I or CG), 2 for double masking (P, OA), 4 studies for single masking (P or OA), and 1 trial opted for an open label approach. Earlier trials incorporated 2 to 3 intervention domains, typically combining physical exercise with either cognitive training or dietary guidance. Later studies expanded their multidomain approaches, integrating further domains such as social engagement, cardiovascular risk management, meditation, or sleep hygiene education. Physical exercise emerged as the most frequently utilized component of intervention, present in all 12 RCTs, with most studies utilizing moderate-to-high intensity progressive training approaches, and only 1 RCT opted for low intensity (Table 1). Ten RCTs incorporated cognitive training in their intervention domains with varying intensities and frequency along with dietary guidance varying between Mediterranean-like and a ketogenic diet, through general healthy dietary advice. Five RCTs incorporated guidance on social activities, ranging from suggestions such as dancing or participating in church events to integrating social engagement within physical exercise or cognitive training domains. Six RCTs employed a combination of individual and group sessions for intervention domains (Table 1, e.g., dietary guidance, physical exercise, and cognitive training) using personalized sessions to tailor the intervention (e.g., addressing weight loss or malnutrition) and group formats to foster socialization and peer support. In contrast, 2 trials relied solely on group sessions (excluding the individually tailored home-based exercises commonly included across studies), while 3 RCTs did not specify how the interventions were delivered. Four trials explicitly targeted cardiovascular or metabolic risk factor management as a distinct intervention domain, while 3 included advice on sleep hygiene, stress management, or meditation. Few RCTs (eg, MET-FINGER, MAPT, MIND-AD_mini_)[17,22,23] specified that the different intervention components were gradually introduced, to facilitate adherence, while this information is not reported for other trials. The transition toward targeting multiple risk factors through multidomain interventions reflects a growing emphasis on comprehensive, data-driven strategies to enhance cognitive health outcomes.

### 3.3 Outcome measures

The heterogeneity of the interventions and the varying designs across the RCTs, including target groups and outcome measures, provided limited possibility to synthetize the outcomes of RCTs. Thus, a narrative synthesis was chosen to describe the results. Apart from the Multimodal Preventive Trial for Alzheimer's Disease (MIND-AD_mini_), which was a feasibility trial, and the Lifestyle Intervention Program for Cognitive Impairment RCT, where change in retinal amyloid burden was the main outcome, all RCTs had changes in cognitive measures as their primary outcome. Cognitive assessment tools ranged from various validated rating scales to composite scores commonly used in clinical practices such as the Alzheimer’s Disease Assessment Scale - Cognitive (ADAS-Cog), the Neuropsychological Test Battery (NTB), the Clinical Dementia Rating scale (CDR), the Montreal Cognitive Assessment (MoCA), the Mini Mental State Examination (MMSE), the Alzheimer Disease Cooperative Study Preclinical Alzheimer Cognitive Composite (ADCS-PACC), the Rey Auditory Verbal Learning Test (RAVLT), or the Stroop color and word test (SCWT). Despite variability in these cognitive assessment tools, core domains such as memory, attention, language, executive function, processing speed and orientation were consistently evaluated, offering a comprehensive view of cognitive function in individuals at risk for dementia. In addition to cognitive outcomes, structural and functional neuroimaging, blood-based biomarkers, and changes in lifestyle indices were often employed as secondary outcome measures, providing a multidimensional evaluation of intervention effects.

### 3.4 Multidomain lifestyle interventions combined with nutraceuticals

#### 3.4.1 Lifestyle interventions with Omega-3 polyunsaturated fatty acids

Omega-3 polyunsaturated fatty acids (PUFA) have been tested in RCTs with cognitive endpoints in older individuals with or without established dementia diagnosis [[24], [25], [26], [27]]. For some studies, findings pointed towards beneficial effects on cognitive outcomes (visuospatial learning, episodic memory, verbal recognition) in younger-old individuals with age-related cognitive decline (>55 years)[24]; however, no cognitive improvements were found either after shorter (6 months)[26] or longer (24 months)[26] supplementation of docosahexaenoic acid (DHA) and eicosapentaenoic acid (EPA) in cognitively healthy older adults (>65 years, or >70 years respectively). A recent meta-analysis included 24 trials, with 9660 participants, ranging from cognitively normal to MCI. The studies varied in intervention length (3 to 36 months), and in PUFA supplement composition and dosage. Overall, the intake of omega-3 polyunsaturated fatty acids was not associated with significant cognitive changes [28]. However, the existing methodological heterogeneity among studies might prevent proper assessment of the efficacy of omega-3 supplementation, in people with and without cognitive symptoms [28,29].

Supplementation with omega-3 polyunsaturated fatty acids was among the first combination trials conducted, possibly due to their excellent safety, ease of administration and high combination potential with multiple domains of lifestyle intervention [15,30]. The Multidomain Alzheimer Preventive Trial (MAPT, clinicaltrials.gov registration nr: NCT00672685) was the largest 4-arm superiority combination, phase III RCT, including 1680 participants, 70 years and older, with subjective cognitive complaints. Participants were randomized to 1 of 4 groups: lifestyle intervention (physical activity advice, cognitive training, and nutritional consultations) + omega-3 supplementation (daily dose of 800mg DHA and 250mg EPA), lifestyle intervention + placebo, lifestyle control (information sessions on lifestyle) + omega-3 supplementation, or lifestyle control + placebo (Table 1). Primary efficacy outcome was change in the composite *Z* score calculated from the combination of the Free and Cued Selective Reminding Test, 10 MMSE orientation items, the Digit Symbol Substitution Test score from the Wechsler Adult Intelligence Scale, and the Category naming test [15]. Although no significant intervention effect on the primary outcome was reported, positive results were found in sub-group and post-hoc analyses, particularly in individuals with higher dementia risk (increased Cardiovascular Risk Factors, Aging, and Incidence of Dementia, or CAIDE dementia risk score), and in participants classified as amyloid-β–positive via brain PET scan with florbetapir. Cognitive benefits were also seen when pooling all participants who received the multidomain intervention (Table 2) [31].

**Table 2:** Short summary of published results regarding outcomes from the identified RCTs. Abbreviations: ADAS-Cog, Alzheimer’s Disease Assessment Scale Cognitive; ADCS-PACC, Alzheimer’s Disease Cooperative Study—Preclinical Alzheimer Cognitive Composite; CAIDE, Cardiovascular Risk Factors, Aging and Dementia; CDR- SOB, Clinical Dementia Rating Sum of Boxes; CGIC, Clinical Global Impression of Change; CI, Confidence interval; CT, Cognitive training; Cv, Cardiovascular; D, diet; EGCG, Epigallocatechin gallate, FDR: false discovery rate; HDI- Healthy Diet Indicator; LDL, Low density lipoprotein; LIBRA, Lifestyle for Brain health; M, Meditation; MCI, Mild cognitive impairment; MEDAS, Mediterranean Diet Adherence Screener; MMSE, Mini mental State Examination; MRI, Magnetic resonance imaging; PE, Physical exercise; PsE, Psychoeducation; S, Stress management; SA, Social activities; SH, Sleep hygiene; Vit, Vitamin.

NCT number	Study Acronym	Intervention (lifestyle plus treatment arm)	Results on primary outcome	Results on other outcomes	Refs
NCT00672685	MAPT	PE, CT, D, Cv + Omega3	No intervention effect on the primary (cognitive) outcomes	•Less decline in 10 MMSE orientation items in lifestyle+ nutraceutical supplement arm versus placebo arm (adjusted p = .036) •In the amyloid β positive sub-population, there was a trend for the combined intervention benefit on the change in composite cognitive score at 12 (adjusted p = .1144, 95 % CI = [0.0136 to 0.3699]) and 36 months (adjusted p = .0690 95 % CI = [0.0190 to 0.5446]) • The combined intervention group showed reduced cognitive decline, compared to placebo, among participants with a baseline CAIDE score ≥6 (p=.023)	[15,31]
NCT01219244		PE, CT + Omega3-VitE	No intervention effect on the primary (cognitive) outcomes	•Reduced atrophy in frontal, parietal and cingulate cortices of MCI patients following combined lifestyle+ nutraceutical intervention •No changes in inflammatory, metabolic or vascular parameters	[30]
NCT02409238	IRMCI	PE, D + Metformin	NA	NA	
NCT03382353	EMuNI	PE, CT, D + Tramiprosate	•Multidomain intervention + Tramiprosate improved on attention-executive composite score, compared to control arm (p = .002). •No significant differences in other cognitive endpoints	•No significant effects on MRI structural measures, but improvement in functional connectivity of the fronto-parietal executive network in the multidomain intervention, compared to the other arms. • Beneficial effects for the multidomain intervention, compared to control, for depressive symptoms (p = .011), and sled-reported memory complaints (p = .013).	[58]
NCT02741804		PE, CT, D, M, SA, SH +BBH-1001	NA	NA	
NCT02808676	SYNERGIC	PE, CT +Vit D3	•At 6 months, all active arms with aerobic-resistance exercise, regardless of the addition of cognitive training or vitamin D, improved ADAS-Cog-13 when compared with control. Compared with exercise alone, exercise and cognitive training improved the ADASCog-13. No significant improvement was found with vitamin D. The multidomain intervention (exercise + cognitive training + vit D) improved the ADAS-Cog-13 compared with control. •ADAS-Cog-Plus was not modified by any combination of interventions.	•No significant correlations between change in functional brain connectivity and change in cognitive or physical function. •Some significant differences in specific cognitive subdomain tests observed between intervention arms and control group.	[37,38]
NCT03249688	MIND-AD_mini_	PE, CT, D, Cv, SA + Souvenaid	•In the lifestyle intervention arm, 78.1 % adhered to at least 2 out of 4 intervention domains (attending ≥40 % of sessions) • In the lifestyle + medical food group 87.1 % of participants were overall adherent in 2 out of 4 lifestyle interventions (consuming ≥60 % medical food)	•Good adherence to healthy lifestyle and improved dietary indexes in the intervention + medical food group (HDI: p < .042 and MEDAS: p < .007) compared to control group •The lifestyle + medical food intervention arm had a significantly lower likelihood for decreasing cognitive-functional level (ie, increasing CDR-SOB) compared with control group •No statistically significant differences between either intervention arm or control in global CDR score	[44,77]
NCT04606420		PE, D, S+ Multi-nutrient supplement	•Significant differences between the intervention group and the control group in cognition and function in the CGIC (p = .001), CDR-SB (p = .032), and CDR Global (p = .037) tests • Borderline significance in the ADAS-Cog test (p = .053)	•Plasma Aβ42/40 ratio significantly differed between intervention and control group (p= 0.003) •LDL-cholesterol decreased in the intervention group, and changes correlated with lifestyle index at 20 weeks (p < .0001, correlation: 0.678) •Significant change in microbiome taxa composition	[60]
NCT03978052	PENSA	PE, CT, D, PsE, SA + EGCG	No intervention effect on the primary (cognitive) outcome	• Significant cognitive benefits assessed by the PACC-exe Z score (P=.005), the Memory Composite Z score (p=.022) and the Semantic Fluency Test (p=.007) were found in the lifestyle+ EGCG group after 15 months (three-month washout period) when compared to participants receiving lifestyle+ placebo intervention. • Both structured lifestyle intervention groups showed significant reductions in LIBRA index scores relative to the control group (lifestyle+EGCG: p =.012; lifestyle+placebo: p =.049) • EGCG supplementation did not affect brain structure nor blood AD biomarkers when compared to placebo • Lifestyle+EGCG group outperformed lifestyle+placebo in improvements in adherence to Mediterranean diet (p=.017) following a three-month washout period.	[51]
NCT05256199	FINGER-NL	PE, CT, D, Cv, SA, SH, S+ Souvenaid	Ongoing	Ongoing	
NCT05109169	MET-FINGER	PE, CT, D, Cv, SA+ Metformin	Ongoing	Ongoing	
NCT05894954	EVANTHEA	PE, CT, D, SH, S+ Personalized intervention	Ongoing	Ongoing	

A smaller RCT enrolling 45 MCI patients (a substudy within a larger study, clinicaltrials.gov ID NCT01219244) has also assessed the combined effect of lifestyle intervention (physical and cognitive domains) with omega-3 supplementation (daily dose of 880mg DHA and 1320 mg EPA supplemented with 15mg vitamin E) [30]. Participants were assessed over 6 months (Table 1). Changes in composite *z* scores of executive function, memory, sensorimotor speed, and attention (based on Auditory Verbal learning test, forward and backward digit spans, verbal fluency, trail making test part A and B, Stroop Color-Word test) were assessed, with no significant differences among the study arms. This pilot study found significantly reduced atrophy in frontal, parietal, and cingulate cortices following combined intervention compared with the control arm (i.e., omega-3 fatty acid supplementation and non-aerobic exercise, Table 2).

While no significant changes were found in cognitive performances after a 6-month[30] or a 36-month-intervention[15] with omega-3 fatty acids combined with multidomain lifestyle intervention, these trials have provided important knowledge on the need to establish the right window of opportunity for such interventions, the right target populations, as well as determination of the optimal dose and treatment duration.

#### 3.4.2 Lifestyle intervention combined with BBH-1001

BBH-1001 is a brain health supplement containing a combination of various nutrients: turmeric (125mg), fisetin (16.65mg), green tea leaf extract (17.5mg), EPA (75mg), DHA (150mg) and vitamin D3 (250IU). Fisetin, a naturally occurring flavonoid with senolytic activity, present in various fruits, vegetables, and teas,[32] has been used alone or in combination in several clinical trials targeting a variety of conditions. The impact of this micronutrient supplement combined with a comprehensive low-intensity lifestyle intervention program was tested on retinal amyloid levels in patients with MCI (NCT02741804, Table 1). Cognitive functioning was assessed as a secondary outcome (change in NTB scores). Participants of this 18-month single-masked 2-arm trial received the BBH-1001 supplement (4 softgels per day) combined with lectures on 6 lifestyle domains (nutrition, physical activity, meditation, sleep hygiene, cognitive activity, and social engagement). The status of this trial is currently unknown, and no published results have been identified.

#### 3.4.3 Lifestyle intervention combined with vitamin D

Vitamin D is involved in the regulation of calcium and phosphorus metabolism, with growing evidence suggesting it also exerts neuroprotective effects via antioxidative mechanisms while also inhibiting neuroinflammation [33]. Vitamin D deficiency has been previously associated with alteration in cognitive processes and dementia in preclinical and some clinical studies [33,34]. Over 10 clinical trials in individuals with cognitive impairment have been completed so far, with several still ongoing, testing the effects of vitamin D alone or in combination with other nutraceuticals. The SYNERGIC double-blinded RCT (SYNchronizing Exercises, Remedies in Gait and Cognition) evaluated the synergistic potential of vitamin D supplementation combined with cognitive and physical training on cognitive function and mobility in older adults with MCI (NCT02808676, Table 1). While this trial was terminated early due to the COVID-19 pandemic, and did not meet the participant recruitment target, the trial protocol and results have been published [35,36]. A total of 175 participants diagnosed with MCI were randomized into 5 study arms (with each arm comprised of 34 to 37 participants): 1) physical exercise (aerobic exercise and resistance training) + cognitive training + vitamin D (10.000IU/week), 2) physical exercise + cognitive training + placebo, 3) physical exercise + sham training + vitamin D, 4) physical exercise + sham training + placebo, or 5) balance and toning + sham training + placebo. The interventions were 3 times per week over a 20-week period. The primary outcomes included the ADAS-Cog13 and the Plus variant, measured at baseline, at 6 months and after 12 months (post-intervention follow-up). In a subset of participants functional brain connectivity was also assessed, but no significant correlations with lifestyle intervention effects were found. ^37^Although the study was underpowered due to its early termination and some of the comparisons did not withstand false discovery rate (FDR) correction, physical exercise with cognitive training significantly improved ADAS-Cog-13 scores, driven by improvements in episodic memory, attention, and orientation [38]. These changes remained significant at the 12-month follow-up as well [38]. ADAS-Cog-Plus did not improved significantly by any combination of the interventions [38]. While physical exercise alone or combined with vitamin D supplementation did not improve cognition,[38] it still emerged as the primary contributor to improvements in functional brain connectivity (Table 2) [37].

#### 3.4.4 Lifestyle interventions combined with Souvenaid

Souvenaid® is a multi-nutrient formulation comprising DHA, EPA, uridine monophosphate, choline, B-vitamins (B12, B6, folic acid), vitamin C, vitamin E, phospholipids, and selenium. Preclinical studies have demonstrated the neuroprotective properties of this combination of nutrients, suggesting a potential damage reduction in neurological conditions associated with AD [39,40]. A pilot clinical trial involving 225 patients with mild AD dementia evaluated the effects of Souvenaid® (Fortasyn Connect) over a 12-week period. The results of cognitive tests showed improved delayed recall, with a sub-analysis indicating that the benefits were most pronounced when supplementation began early in the prodromal stage of AD [41]. A 2-year clinical trial, with optional 1-year double-blind extension, tested Souvenaid® in 311 prodromal AD patients (LipiDiDiet, Netherlands clinical trial registration number NL1620)and reported a significant slowing of cognitive-functional decline (CDR-sum of boxes, CDR-SB), and attenuated hippocampal atrophy at 2 years. No significant effect was reported on the primary outcome (5-item NTB), but the cognitive decline in the study population was less than expected, reducing statistical power [42]. Data from the 3-year time point confirmed the cognitive benefits of Souvenaid®, in terms of primary and secondary endpoints (i.e., 5-item NTB score, NTB memory domain, CDR-SB) and reduction of brain atrophy [16]. Clinically, these results were estimated to translate into a delay of 7 to 10 months in disease progression, based on analyses using various time-component tests from the 2-year data [43].

The 6-month multinational, proof-of-concept Multimodal Preventive Trial for Alzheimer's Disease (MIND-AD_mini_), conducted within the World-Wide FINGERS (WW-FINGERS) network, investigated the feasibility of FINGER-based lifestyle intervention (nutritional guidance, physical activity, cognitive training, social activities, and monitoring of vascular and metabolic risk factors) with or without Souvenaid®, compared with standard of care (NCT03249688) in 93 patients with prodromal AD. The primary focus of this trial was on feasibility outcomes, while adherence to healthy lifestyle changes was examined as a secondary outcome [17,44]. Change in the CDR-SB was also evaluated as exploratory outcome. The study showed good feasibility and excellent adherence to the combined intervention (Table 2), which seemed to additionally have a benefit on CDR-SB. These positive results pave the way to larger trials validating the clinical efficacy of the combination of Souvenaid® + FINGER multidomain lifestyle intervention in people with prodromal AD.

The larger 2-year Dutch FINGER-NL trial (NCT05256199, Table 1) is currently investigating the effects of combined Souvenaid® and multidomain lifestyle intervention in 1210 individuals at risk of dementia, due to the presence of 2 or more modifiable risk factors plus either SCD or family history of dementia [45]. FINGER-NL is also part of the WW-FINGERS network [13]. Integrating lessons learned during the COVID-19 pandemic, it adapted a hybrid design involving a digital intervention platform with custom-made training materials (intervention group) or general lifestyle health advice (control group). The trial was estimated to be completed in 2025, with results on its primary outcome—change in NTB-based composite scores—anticipated to be available early 2026.

#### 3.4.5 Lifestyle intervention combined with Epigallocatechin gallate

Epigallocatechin gallate (EGCG) is a flavanol from green tea, with a good safety profile and broad mechanism of action including antioxidant activity, protection against neuroinflammation, disaggregation of tau, along with potential regulation of insulin signaling [46,47]. More than 100 clinical trials for a broad range of therapeutic areas have been conducted, including for various malignant tumors, obesity, and neurological conditions such as multiple sclerosis, AD, Down syndrome and Parkinson’s disease. Outcomes have been mixed, possibly also related to EGCG’s erratic bioavailability [48]. Because EGCG can easily undergo modifications or inactivation by concomitant milk consumption, ingestion after a fasting period, with at least 30 min prior to breakfast, has been recommended [48]. A 12-month clinical trial, enrolling 84 patients with Down syndrome, has shown improvements in visual recognition memory, inhibitory control, and adaptive behavior following EGCG (9 mg/kg/daily dose) combined with cognitive training [49]. The efficacy of EGCG in combination with multimodal intervention (dietary guidance, physical exercise, psychoeducation, social activities and cognitive training) in slowing down cognitive decline was assessed in the PENSA study in *APOE-ε4* carriers with SCD (NCT03978052, Table 1) [50]. This randomized control trial enrolled 129 *APOE-ε4* carriers, who were allocated to 1 of 4 treatment arms: EGCG (300-500mg/day) combined with a multimodal intervention; placebo combined with a multimodal intervention; EGCG with lifestyle recommendations; and placebo with lifestyle recommendations [51,13,52]. . The PENSA study is part of the WW-FINGERS network,[13] with a multimodal intervention adapted from the FINGER trial [10]. Following a 12 months intervention, no statistically significant change has been observed in the study primary outcome - change in global cognition assessed by modified ADCS-PACC, with inclusion of tests of executive functions (PACC-exe) (Table 1). Nonetheless, exploratory analysis indicated that participants receiving the combined lifestyle intervention and EGCG were 2.6 times more likely to show cognitive improvements, compared to those receiving lifestyle intervention plus placebo. This group also demonstrated greater improvements in insulin resistance and mediterranean diet adherence[51] compared to those receiving lifestyle and placebo intervention, and both lifestyle intervention groups improved in physical fitness. Notably, significant cognitive benefits in the multidomain intervention + EGCG group were found after a three-month washout period, when compared to participants receiving multidomain intervention + placebo. Furthermore, all structured lifestyle intervention groups - whether paired with EGCG or placebo- outperformed those given recommendations alone in terms of improvements in some of the cognitive measures [51]. Overall the study indicated the feasibility and potential therapeutic benefits of combination interventions for a population at risk of dementia [52].

### 3.5 Multimodal lifestyle interventions combined with pharmaceutical compounds

#### 3.5.1 Lifestyle intervention combined with Metformin

Increasing evidence highlights that pharmacological strategies for decreasing insulin resistance and preventing T2D may also help reduce the risk of cognitive impairment [53,54]. Metformin, the first-line treatment for T2D, has been identified as a promising repurposed pharmaceutical agent to prevent or delay cognitive impairment. In an open-label trial, metformin (750mg/day) was administered in combination with multidomain intervention (physical activity and dietary modification) to individuals with MCI and T2D or prediabetes. The trial aimed to enroll 360 patients, with a 2-year follow-up (clinicaltrials.gov registration nr: NCT02409238, Table 1), but it did not meet its recruitment goals and was terminated due to a combination of lack of funding, retirement of the main investigator, and the COVID-19 pandemic.

The ongoing METformin and FINGER Intervention to Prevent Cognitive Impairment and Disability in Older Adults at Risk for Dementia (MET-FINGER) trial, also part of the World-Wide FINGERS network, is an innovative 2-year multinational phase-IIb RCT (clinicaltrials.gov registration nr: NCT05109169, Table 1) combining metformin with multimodal lifestyle intervention [22]. This combination trial bridges the gap between pharmacological and non-pharmacological strategies for dementia prevention and uses a novel precision prevention approach, as it targets an *APOE-*ε4 enriched population of 600 older adults (60–79 years) at increased risk of dementia, identified via assessment of vascular risk factors and cognitive screening [11,22]. The structured multimodal lifestyle intervention (SMLI, an optimized FINGER model) is combined with metformin when appropriate (active arm), and compared with self-guided lifestyle intervention (control arm). Participants allocated to the SMLI and at increased risk of T2D are further randomized to additionally receive metformin 2000mg/day, metformin 1000mg/day, or placebo (double-blind), with a trial-within-trial study design. This pragmatic approach mirrors potential real-life scenarios where disease-modifying treatments are given to specific at-risk populations for whom they are most effective. The primary outcome is change in global cognition (NTB overall score). Recruitment is expected to be completed by the end of 2025. This trial is expected to provide critical insights for developing and refining innovative dementia prevention strategies, focusing on delivering the most effective solutions to the right individuals at the optimal time.

#### 3.5.2 Lifestyle intervention combined with Homotaurine (Tramiprosate)

Tramiprosate and its derivative valiltramiprosate (ALZ-801) are small molecules reported to inhibit Aβ42 aggregation into toxic oligomers, by stabilizing Aβ42 through binding at specific sites including Lys16, Lys28 and Asp23 [55]. A phase III clinical trial of Tramiprosate in AD patients failed to meet the primary efficacy endpoints, but a subgroup analysis revealed significant cognitive improvements, measured by ADAS-Cog scores, and a positive trend on CDR-SB, in *APOE*-ε4 homozygous participants with milder cognitive decline [56]. Tramiprosate efficacy combined with multidomain intervention was tested in the EMuNI RCT recruiting patients with SCD (NCT03382353, Table 1) [56]. Participants were randomized to 1 of 3 study arms: the active control intervention arm (n=41) received educational training, the partial intervention arm (n=45) received 100mg/day of Tramiprosate along with nutritional guidance, and the multilevel intervention arm (n=48) received 100mg/day of tramiprosate over the course of a year combined with nutritional guidance, physical exercise, and cognitive training [57]. Outcome measures included the RAVLT delayed recall test and composite scores of global cognition, memory, attention, executive and visuospatial scores, as well as structural and functional imaging [57]. The intervention adherence reported was 80 % or higher for all arms, and one of the primary cognitive outcome measure (attention-executive composite score) indicated a beneficial effect for the multilevel intervention, compared to the control group (Table 2) [58].

### 3.6 Other approaches

Our search strategy additionally identified the ongoing Precision Medicine Approach for Early Dementia & Mild Cognitive Impairment (EVANTHEA) trial (NCT05894954, Table 1), described as a pragmatic, randomized, controlled trial to evaluate the effectiveness of a precision medicine treatment approach for early dementia and MCI. The trial protocol seems to have been based on a published case report series of 10 patients [59]. EVANTHEA aims to recruit 72 participants aged 45 to 76 years, randomized to a 9-month precision medicine treatment approach or a 9-month standard-of-care treatment. The description of the combination intervention lists a very broad range of supplements, hormones, medications, and other lifestyle and nonpharmacological components that are meant to be tailored to a broad range of laboratory tests and other participant characteristics. It is, however, unclear how this will be implemented.

Results from a recent RCT investigating the effect of multidomain lifestyle intervention with multi-nutrient supplements—multivitamins, Omega-3 fatty acids (1680 mg), curcumin (800 mg), vitamin C (1g), vitamin B12 (500 mg), CoQ10 (200 mg), lion's mane (2g), Super Bifido Plus Probiotic (1 tablet/day), and magnesium (144mg)— in MCI and early AD patients (NCT04606420, Table 1) has been recently reported [60]. The trial had not met its original recruitment aim of 100 participants and randomized 51 individuals to either the lifestyle intervention (physical exercise, dietary guidance, stress management) plus supplements or control (usual care) arms, with the aim of offering all individuals in the control arm a crossover to intervention after 20 weeks (i.e., individuals in the lifestyle arm continue the intervention for 40 weeks in total). Results were published after the first phase of the 20-week randomized controlled part of the study, with significant changes reported (albeit with 1-tailed statistical tests) on most primary cognitive outcomes on CGIC, CDR-SB and CDR Global tests, along with borderline significance in the ADAS-Cog test (Table 2). Further significant changes in plasma amyloid β42/40 ratio, along with LDL cholesterol levels were also reported [60].

## 4 Discussion

### 4.1 Key methodological points

Recently, numerous modifiable risk factors of dementia have been identified, affecting dementia risk to different extents throughout the lifespan [8]. The long preclinical stage of AD, which precedes cognitive impairment and the onset of dementia, offers a valuable window of opportunity for prevention [7]. Due to the complex and multifactorial nature of ADRD, precision medicine and combination therapy approaches, integrating lifestyle interventions and pharmacological treatments to target multiple disease pathways may be more effective. Combining pharmacological and non-pharmacological interventions has yielded positive results in chronic disorders linked to ADRD (i.e., T2D, cardiovascular disease), and has the potential to promote sustained clinical benefits, reduce possible adverse events of drugs, and improve overall intervention adherence in older adults at increased risk for dementia.

Our systematic review identified a total of 12 RCTs testing combination therapies of pharmacological compounds or nutraceuticals and multidomain lifestyle interventions. These combination RCTs exhibit significant variability in key aspects of their design, such as the type and dosage of agents administered, target populations and stage across the AD continuum, intervention duration, and the composition and intensity of the intervention domains.

About half of the reviewed combination RCTs had published results. While some of the RCTs reported cognitive benefits, there was variability in the findings. For example, in the MAPT trial, while the primary outcome analysis did not show significant between-group differences, improvements were reported in at-risk subgroups when comparing lifestyle intervention plus nutraceutical versus placebo arms [15,31]. In the SYNERGIC RCT, lifestyle intervention improved ADAS-Cog-13, when compared to the control group, albeit without a combined effect observed following vitamin D administration [37,38]. In PENSA trial, combining lifestyle intervention with EGCG showed a trend toward cognitive improvement that became significant after a three month washout, indicating potential long term benefits in *APOE*-ε4 carriers with SCD [51]. In the EMUNI trial, a significant intervention effect on the attention- executive composite score was reported in the multidomain intervention group when compared to control group following 12 months of tramiprosate treatment [58]. Furthermore, a small trial in MCI and early AD reported some benefits in cognitive/functional outcomes following a combination of multi-nutrient supplementation and lifestyle intervention when compared to the usual care control group [60].

#### 4.1.1 Evidence-based selection of pharmacological agents for combination therapies

A range of pharmacological formulations were integrated with lifestyle interventions in combination therapies. Supplements and medical food such as omega-3 compounds, vitamin D, and Souvenaid, which are generally available over the counter, have been suggested to have beneficial effects on cognition. However, no robust intervention benefit was found for omega-3 or vitamin D supplementation in combination with multidomain lifestyle interventions. This is in line with the 2019 WHO guidelines for cognitive decline and dementia risk reduction, which noted the lack of sufficient evidence for polyunsaturated fatty acids as a preventive nutraceutical [61]. On the other hand, Souvenaid has shown significant cognitive-functional benefits in prodromal AD [43].

With aging being the leading risk factor for ADRD, major efforts are underway to therapeutically target the processes that go awry with aging that have also been implicated in the pathophysiology of ADRD, including inflammation, impaired autophagy, mitochondrial dysfunction, vascular dysfunction, epigenetic changes, and synaptic loss. And given the same biological aging mechanisms underlie common chronic diseases of aging (eg, cardiovascular diseases, metabolic diseases), it is worth testing repurposed drugs already approved for these indications for dementia prevention. Drug repurposing approaches provide the added advantage of established safety profiles. Similar approaches have enabled the development of successful therapies for, e.g., cancer, HIV, or Parkinson`s disease [62,63]. The ongoing MET-FINGER trial is among the first examples of a multidomain lifestyle plus repurposed drug combination therapy. Given the link of T2D with cerebrovascular disease and AD, and the potential beneficial pleiotropic effects, including anti-inflammatory, neuro-protective, and anti-senescence effects, metformin is a very suitable candidate for drug-repurposing in dementia prevention. Other T2D medications, e.g., glucagon-like peptide-1 (GLP-1) agonists, may also be relevant for future combination therapies [64].

Novel disease-modifying therapies (DMTs) also represent promising candidates for integration into combination therapies. Anti-amyloid β antibody therapies (lecanemab and donanemab) are currently approved by several regulatory authorities in the EU and elsewhere for treatment of MCI and mild dementia due to AD and notably, the Food and drug administration agency (FDA) has recently approved the subcutaneous formulation of lecanemab for weekly maintenance dosing following initial intravenous therapy [65]. However, eligibility for these DMTs is limited, and *APOE-ε4* homozygous patients have a higher risk for serious side effects [2,66,67]. While their current availability and mode of administration (infusion) is not optimal for integration with lifestyle interventions, this may change if equivalent efficacy can be achieved through less frequent dosing or subcutaneous administration. Of note, efficacy and safety in preclinical AD and more advanced stages of AD dementia have not been established [66]. An evidence-based, expert consensus process could speed up the search for candidate DMTs for combination therapy RCTs. Global drug selection initiatives have been already developed for, e.g., Parkinson’s disease. Selection criteria for novel or repurposed pharmacological agents for combination therapy RCTs will likely require a multilayered approach, considering, e.g., their probable added benefit, safety profile, and feasibility for combination with multimodal lifestyle interventions.

#### 4.1.2 Identification of target populations

Effective interventions require identification of the at-risk target groups that are more likely to benefit. Personalized approaches could be implemented based on individual risk profiles, including, e.g., age, genetic predisposition (such as *APOE-ε4* allele), family history of memory impairment, comorbidities, and other specific modifiable risk factors, with interventions tailored and delivered in a manner that can be sustained by the individual. Four of the combination RCTs we identified considered the presence of SCD as enrichment strategy (Table 1). All FINGER-based combination RCTs (MIND-AD_mini_, MET-FINGER, PENSA, FINGER-NL) included older adults (≥60 years) with modifiable risk factors, identified in some RCTs based on validated multifactorial dementia risk scores such as CAIDE [68]. Additionally, dementia risk factors such as T2D, or genetic risk such as the *APOE-ε4* allele were also reflected in the design of MET-FINGER, IRMCI and PENSA RCTs. In MET-FINGER, participants are randomized in 2 steps, so only those who can benefit from metformin -because of central obesity or impaired glucose metabolism but no T2D – are randomized to receive the experimental drug (metformin or placebo) [22]. Ultimately, the goal is to optimize the target group selection, with higher potential for therapeutic response, ensuring also adequate statistical power to assess the experimental intervention in a given RCT. Overall, recruitment strategies need to be further developed for efficient enrollment of study participants from various sources including hospital settings (such as memory clinics, brain health clinics), the general population, and readiness cohorts with available data for pre-screening. Ensuring diversity across ethnic, sociodemographic, and economic backgrounds remains a key priority, which has been so far inadequately addressed in combination trials. Multinational and multisite trials, such as MET-FINGER, provide the possibility for recruitment of more diverse populations [22]. In existing platforms and networks, such as World-Wide FINGERS, where lifestyle-based multidomain intervention trials are tested in populations from diverse racial and ethnic groups (e.g., Caucasians, Afro-Americans, Mestizo, Malay, Indian, Chinese)[[69], [70], [71]], understanding the variations in key factors affecting cognitive trajectories (e.g., education, socio-economic status, genetic make-up), can also facilitate development of multinational combination trials with better representation of populations often not included in ADRD RCTs.

#### 4.1.3 Biomarkers and biorepositories

Recent biomarker developments could greatly facilitate implementation of easily accessible (e.g., blood-based) markers for disease processes in RCTs to select suitable target populations and/or investigate responses to combination therapies;[72] a notable example is the recent FDA approval of the Lumipulse G blood test, which measures pTau217/ß-Amyloid 1-42 plasma ratio and offers a less invasive alternative to PET scans for AD diagnostics [73]. Large ongoing international projects are currently testing the real-world implementation of early detection tools for AD (eg, AD-RIDDLE) [74]. The identification and validation of novel biomarkers and multi-marker risk and disease biosignatures, along with creating the framework for large biorepositories, are essential for advancing combination RCTs. As RCTs become increasingly complex, the interpretation of biomarker findings may also be challenging, particularly in combination and add-on trial designs. Robust biomarker frameworks will be critical to guide timing and personalization efforts of multidomain interventions [7].

In addition to biomarkers of AD pathology (Aβ42/40 ratio, ptau217, etc.), emerging biomarkers that reflect the biological processes of aging are highly relevant for prevention trials given aging is the primary risk factor for sporadic AD. Current progress in biomarker development encompasses the full spectrum of aging biology, including inflammation, cellular senescence, synaptic dysfunction, vascular dysfunction, aberrant proteostasis, mitochondrial oxidative stress, metabolic dysfunction, and epigenetics [75]. As these biomarkers become validated, future studies can leverage these for enriching trial populations that would most likely benefit based on the mechanism of action of the intervention.

#### 4.1.4 Study design for combination RCTs

The design of lifestyle intervention combination RCTs for ADRD, albeit recent, has advanced considerably in the last decade. As risk factors for ADRDs were better characterized, study designs evolved from early trials incorporating 2 or 3 intervention components (eg, MAPT or EMuNI), to more complex multidomain approaches (eg, FINGER-based). With this shift, key design elements such as intervention intensity and duration, level of personalization, sample size calculations, and participant allocation have also become increasingly complex. Earlier studies typically featured shorter interventions with smaller sample sizes and were often underpowered, which likely contributed to the lack of observed intervention benefits in some RCTs. More recently, intervention durations have spanned between 12 and 36 months, with larger sample sizes. Nonetheless, heterogeneity in intervention intensity, due to different doses or variations in intensity and mode of delivery per lifestyle domain, may influence both intervention effectiveness and participant adherence.

**Fig. 2:**
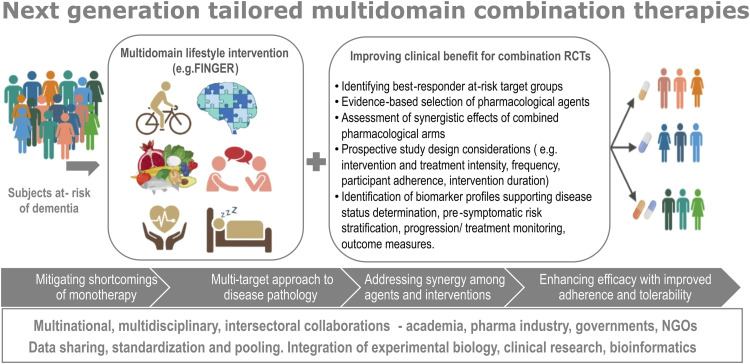
Schematic representation of the study design recommendations for improved clinical benefit for the next generation tailored multidomain combination therapies(Created with BioRender.com). Abbreviations: FINGER, Finnish Geriatric Intervention Study to Prevent Cognitive Impairment and Disability; NGO- Non governmental organization; RCT, randomized controlled trial.

Potential interactions between intervention components may further contribute to the heterogeneity of RCT findings. These challenges underscore the need for more standardized combination RCT methodologies. Despite substantial progress, further innovative study designs are needed to better capture long-term intervention effects, accommodate individual variability in risk profiles, and improve scalability for real-world applicability (Fig 2). Crucially, the impact of an individual's risk or disease profile on shorter- and longer-term effects of interventions remains unclear.

The integration of the right pharmacological treatments with the most suitable lifestyle interventions at the appropriate risk or disease stages needs to be systematically explored across the AD continuum, to leverage potential synergistic and additive therapeutic effects, and minimize risk of adverse events. Statistical considerations are critical in optimizing study design. In combination RCTs, factorial (or modified factorial) study designs are usually needed to demonstrate the contribution of each intervention (lifestyle or pharmacological) to the combined effect and to understand whether the combination therapy is additive, antagonistic, or synergistic [7]. However, the 4-arm classic 2×2 factorial design requires a larger sample size. Innovative design elements, such as adding new study arms to existing trials or incorporating precision medicine strategies where treatments are tailored to individual participant profiles could increase the effectiveness of an RCT. Additionally, reducing the size of the control group through adaptive designs could improve study efficiency and ethical feasibility. Emulated trial alternatives such as using historical or real-world data to model control groups could reduce the need for large standard care groups; however, caveats include potential differences in patient characteristics, data collection methods, standard of care, and other confounding factors. Moreover, combination RCTs could accelerate translation into clinical practice by evaluating multiple therapies simultaneously, optimizing treatment strategies, and identifying synergistic effects more efficiently [7]. By incorporating lifestyle interventions, these combination trials could provide a multidimensional approach to treatment, improving long-term health outcomes. This is particularly important in individuals at risk of dementia, where combining pharmacological and lifestyle strategies may yield greater clinical benefits than either approach alone.

Driven by the need for global collaboration and methodological harmonization of multimodal dementia prevention trials, the FINGER intervention model is now being tested, adapted, and optimized across diverse geographical and cultural contexts. The World-Wide FINGERS network,[13] currently counts 70 participating countries, with a portfolio of at least 22 RCTs completed, ∼25 ongoing or in planning stage. Promoting prospective harmonization of study designs, including consideration of intervention aspects such as intensity, frequency, participant adherence, and duration, along with standardized outcomes and possibilities for data sharing will enable joint analyses and cross-study comparisons. Integrating the identification of biomarker profiles to support risk stratification, disease status determination, monitoring of disease progression and treatment effects will generate robust evidence to guide combination therapies for dementia risk reduction.

### 4.2 Limitations of this review

Based on GRADE guidelines,[76] some limitations were identified. Firstly, studies registered on the major clinical trial databases were searched, leading to a potential bias in the study location, as only trials and articles written in English were included. Secondly, potential publication bias must be acknowledged, as clinical trials with significant results are more likely to be published. Moreover, trials were included regardless of whether they had resulted in any publications. Furthermore, results from these RCT studies may not be generalizable beyond the scope of the specific combination of intervention, administered doses, study population, and duration of the intervention. Thus, the evidence-based classifications are presented in the context of these limitations.

### 4.3 Concluding remarks and future directions

These studies have provided preliminary evidence supporting the efficacy of combination therapy approaches that simultaneously target multiple risk factors and disease processes. Given the progressive nature of ADRD, certain therapeutic targets may be more effectively addressed in different stages of the disease continuum. Timing and sustainability of combination therapies, along with understanding the determinants of intervention response, are thus key factors to be evaluated. Adaptive trial designs, including platform trials or response-adaptive randomization could offer flexibility to allocate participants to promising treatment arms in a dynamic manner based on real-time data from the ongoing trial. Recent biomarker developments will facilitate the implementation of easily accessible (e.g., blood-based) markers for disease processes in RCTs to, e.g., select suitable target populations and/or investigate responses to combination therapies. Innovative adaptive or platform clinical trials integrating pharmacological treatments with multimodal lifestyle interventions, and a one-size-does-not-fit-all precision medicine approach, could substantially contribute to development of effective dementia risk reduction strategies.

## Funding

Open access funding provided by 10.13039/501100004047Karolinska Institute. This work was supported by the European Union (EU) Innovative Health Initiative Joint Undertaking (IHI JU) AD-RIDDLE, under grant agreement No. 101132933; Alzheimer's Drug Discovery Foundation (USA); Alzheimer’s Disease Data Initiative (ADDI); Davos Alzheimer's Collaborative; Gates Ventures (USA); EU Joint Programme—Neurodegenerative Disease Research (JPND) Multi-MeMo grant (Research Council of Finland); Alzheimerfonden (Sweden); Region Stockholm research grant (ALF, Sweden); Center for Innovative Medicine (CIMED) at Region Stockholm (Sweden); Stiftelsen Stockholms sjukhem (Sweden); Swedish research council for health, working life and welfare (FORTE); Gun och Bertil Stohnes Stiftelse (Sweden); the Karolinska Institutet fund for Geriatric Research; Stiftelsen Gamla Tjänarinnor (Sweden); Juho Vainio Foundation (Finland); Finnish Cultural Foundation (Finland); Yrjö Jahnsson Foundation (Finland).

## Open Access

This article is distributed under the terms of the Creative Commons Attribution 4.0 International License (http://creativecommons.org/ licenses/by/4.0/), which permits use, duplication, adaptation, distribution and reproduction in any medium or format, when giving appropriate credit to the original author(s) and the source, providing a link to the Creative Commons license and indicating if changes were made.

## Declaration of generative AI and AI-assisted technologies in the writing process

During the preparation of this work the authors used OpenAI’s ChatGPT in order to improve the readability of the manuscript. After using this tool, the authors reviewed and edited the content as needed and take full responsibility for the content of the published article.

## CRediT authorship contribution statement

**Erika Bereczki:** Writing – review & editing, Writing – original draft, Supervision, Conceptualization. **Francesca Mangialasche:** Writing – review & editing, Writing – original draft, Supervision, Conceptualization. **Mariagnese Barbera:** Writing – review & editing, Writing – original draft, Supervision, Conceptualization. **Paola Padilla:** Writing – review & editing, Writing – original draft, Supervision, Conceptualization. **Yuko Hara:** Writing – review & editing, Writing – original draft, Supervision, Conceptualization. **Howard Fillit:** Writing – review & editing, Writing – original draft, Supervision, Conceptualization. **Alina Solomon:** Writing – review & editing, Writing – original draft, Supervision, Conceptualization. **Miia Kivipelto:** Writing – review & editing, Writing – original draft, Supervision, Conceptualization.

## Declaration of competing interest

The authors declare that they have no known competing financial interests or personal relationships that could have appeared to influence the work reported in this paper.
